# Platelet-Rich Fibrin as a Bone Graft Material in Oral and Maxillofacial Bone Regeneration: Classification and Summary for Better Application

**DOI:** 10.1155/2019/3295756

**Published:** 2019-12-06

**Authors:** Yiping Liu, Xiaolin Sun, Jize Yu, Jia Wang, Peisong Zhai, Siyu Chen, Manxuan Liu, Yanmin Zhou

**Affiliations:** Department of Oral Implantology, School of Dentistry, Jilin University, Changchun 130021, China

## Abstract

Platelet-rich fibrin (PRF) is an autologous platelet concentrate that consists of cytokines, platelets, leukocytes, and circulating stem cells. It has been considered to be effective in bone regeneration and is mainly used for oral and maxillofacial bone. Although currently the use of PRF is thought to support alveolar ridge preservation, there is a lack of evidence regarding the application of PRF in osteogenesis. In this paper, we will provide examples of PRF application, and we will also summarize different measures to improve the properties of PRF for achieving better osteogenesis. The effect of PRF as a bone graft material on osteogenesis based on laboratory investigations, animal tests, and clinical evaluations is first reviewed here. In vitro, PRF was able to stimulate cell proliferation, differentiation, migration, mineralization, and osteogenesis-related gene expression. Preclinical and clinical trials suggested that PRF alone may have a limited effect. To enlighten researchers, modified PRF graft materials are further reviewed, including PRF combined with other bone graft materials, PRF combined with drugs, and a new-type PRF. Finally, we will summarize the common shortcomings in the application of PRF that probably lead to application failure. Future scientists should avoid or solve these problems to achieve better regeneration.

## 1. Introduction

The oral maxillofacial bone is a site of predilection for tumors, inflammation, trauma, and congenital disease. The loss of oral and maxillofacial bone caused by diseases, such as bone removal during tumor surgery, periapical bone destruction due to periapical periodontitis, alveolar bone atrophy after tooth loss, and alveolar cleft, seriously affects the physical and mental health of the patients. Studies on oral and maxillofacial bone regeneration have used many bone graft materials, including autologous bone, allogeneic bone, and synthetic bone graft materials. The use of autogenous bone transplantation, considered as the gold standard, was restricted in bone regeneration due to limited donor supply, a second surgery, chronic pain, and complications at the donor site [1]. Moreover, allografts may transmit disease and synthetic graft substitutes lack the property of osteogenic induction [2, 3].

In recent years, platelet-rich fibrin (PRF) has been considered suitable for oral and maxillofacial bone regeneration [4, 5]. It is considered as the second generation of platelet concentrates because it is made by using a simplified protocol that includes centrifugation of autogenous peripheral blood without any biological agents. In contrast, the first generation of platelet concentrates is PRP which is mainly produced by two-step centrifugation and addition of bovine thrombin and calcium chloride [6]. PRF is a dense fibrin scaffold [7] composed of a fibrin matrix polymerized in a four-molecule structure, and it contains cytokines, platelets, leukocytes, and circulating stem cells [8]. In addition, Dohan Ehrenfest et al. classified platelet concentrates into the following four categories according to their leucocyte and fibrin content: pure platelet-rich plasma (P-PRP), leucocyte- and platelet-rich plasma (L-PRP), pure platelet-rich fibrin (P-PRF), and leucocyte- and platelet-rich fibrin (L-PRF). In this manuscript, PRF refers to L-PRF [9].

PRF contains nearly 97% of platelets and more than 50% of leukocytes in the blood [10]. Among these cells, macrophages can directly promote osteogenesis, which is related to nuclear factor kappa B [11]. Macrophages can also possibly support the activity of bone formation by maintaining local availability of mesenchymal stromal/progenitor cells when recognizing and removing apoptotic osteoblasts to trigger a paracrine loop [12]. Besides, platelets and leukocytes promote bone regeneration by releasing cytokines after activation [6, 13]. The major growth factors in PRF are transforming growth factor-1 (TGF-*β*1), vascular endothelial growth factor (VEGF), bone morphogenetic protein-1 (BMP-1), platelet-derived growth factors (PDGFs), and insulin-like growth factors (IGFs) [6, 14]. TGF-*β*1 may promote new bone formation by stimulating collagen and fibronectin synthesis [15]. Collagen is an important component of bone, while fibronectin can enhance cell adhesion and migration and promote osteogenic differentiation by regulating the Wnt/*β*-catenin signaling pathway [16, 17]. VEGF influences angiogenesis; thus, it is essential for skeletal development [18]. BMP-1 is involved in extracellular matrix deposition [19]. PDGFs and IGF-I have been found to enhance the proliferation and differentiation of osteoblasts [20, 21].

The three-dimensional structure of PRF provides a microenvironment conducive to osteogenesis. The equilateral connective structure of fibers within PRF establishes a thin and flexible fibrin network, which is conducive to cell migration and capture of cytokines [22]. Abundant fibronectin in PRF [23] enhances cell adhesion; an in vitro experiment has shown that human osteoblasts have a higher degree of adhesion to fibronectin than to other extracellular matrix proteins [24]. When the fibrin matrix of PRF undergoes remodeling, cytokines are released gradually [6]. This ensures that the growth factors are released intensively and continuously, and they may therefore enhance cell proliferation during bone formation [25].

In vitro, PRF can improve proliferation, differentiation, migration, and mineralization in cells during bone formation, and the effects vary by the cell type. In many preclinical and clinical studies, PRF alone, PRF combined with other bone graft materials, and PRF combined with drugs promoted oral and maxillofacial bone regeneration in vivo. Some modified PRF graft materials have improved components and structure, or they have eliminated some limitations during application. Finally, we highlight the common shortages during application, and future studies can optimize the preparation process and the experimental design of PRF and further elucidate the mechanism of action of PRF in regeneration, thus leading to better bone quality.

## 2. Osteogenesis-Enhancing Effect of PRF on Stem Cells of Oral and Maxillofacial Origin

PRF may be a potential replacement for osteogenic medium in bone regeneration [26]. In most studies (Table 1 [23, 27–30]), PRF has shown enhancing effects on stem cell proliferation, differentiation, migration, and mineralization during bone formation, but the effects vary by the cell type. Thus, the choice of cell type affects osteogenesis.

Gingival stromal progenitor cells (GSPCs) cultured with PRF increased the expression of the early marker of osteogenic differentiation—core-binding factor subunit-*α*1 (CBF-*α*1)— compared with that in the osteogenic medium culture plate and the negative control group. The highest average CBF-*α*1 expression was found in the PRF treatment group on day 7, whereas the lowest average CBF-*α*1 expression was found in the negative control group on day 21 [27]. In addition, PRF promoted osteogenic differentiation and mineralization of periodontal ligament stem cells (PDLSCs) compared with that in non-PRF groups in another study [28]. Periodontal progenitors are known to be incapable of forming bone or other mineralized tissues in tissue engineering without osteoinduction [31], which also indicated the osteogenic induction effect of PRF. Besides, with respect to osteoblasts, they showed enhanced cell growth and proliferation and higher lactate dehydrogenase value and alkaline phosphatase (ALP) activity when cultured in PRF media compared with Bio-Gide®, which may be due to smoother surfaces of the PRF membrane and abundant cytokines therein [23].

Li et al. [29] reported that PRF enhanced osteogenesis, and the effects were cell-specific, which favored alveolar bone (AB) osteoblasts. In vitro, PRF significantly improved periodontal progenitor cell proliferation and migration when compared with platelet-poor plasma (PPP) and Dulbecco's modified Eagle medium (DMEM), and proliferation occurred earlier in AB osteoblasts than in periodontal ligament (PDL) fibroblasts and dental follicle progenitors cultivated in PRF, suggesting that the effects of PRF were tissue-specific, and they favored AB osteoblasts. Osteoblast activity and mineralized nodule formation evaluated by ALP and alizarin red staining showed higher levels in the PRF group. Moreover, PRF markedly enhanced the expression of the osteoblast differentiation transcription factor and runt‐related transcription factor 2 (RUNX2) and reduced the expression of the mineralization inhibitor, matrix GLA protein (MGP) in cells, preferentially in AB osteoblast progenitors and to a lesser degree in the other cells.

**Figure 1:**
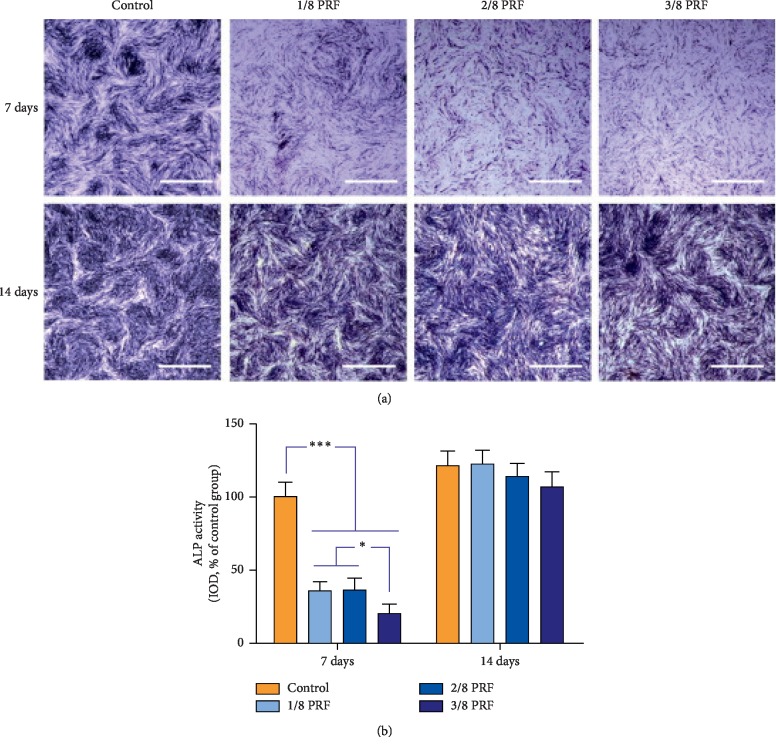
The alkaline phosphatase (ALP) activities of the periodontal ligament stem cells (PDLSCs) from the different experimental groups during a 14-day culture period (*α*-minimum essential medium supplemented with 10% fetal bovine serum, 50 *μ*g/mL ascorbic acid, 10 nm dexamethasone, and 10 mm *β*-glycerophosphate). (a) Representative images for the ALP staining of the PDLSCs cocultured with different doses (1/8, 2/8, or 3/8) of platelet-rich fibrin at different time intervals (scale bar = 200 *μ*m). (b) Data analysis of the ALP activity by means of the integrated optical density (IOD) of representative images (^*∗*^*p* < 0.05; ^*∗∗∗*^*p* < 0.001).

However, another study showed a lower concordance in the effect of PRF on osteogenic differentiation of PDLSCs. In this research, although PRF induced proliferation of human PDLSCs throughout the 7-day incubation period, it suppressed the osteoblastic differentiation of PDLSCs by decreasing the ALP activity (Figures 1(a) and 1(b)) and the gene expression of bone sialoprotein (BSP) and osteocalcin (OC). This can be explained as the effects vary by the cell type [30].

The above experiments indicate that PRF can be used as an osteogenic medium for cultivating GSPCs, PDLSCs, osteoblasts, PDL fibroblasts, and DFSCs, and the enhanced osteogenesis effect may favor the osteoblasts. However, there is a lower concordance in the effect of PRF on PDLSCs.

## 3. Effect of PRF on Oral and Maxillofacial Bone Regeneration in Animal Models

Applications of PRF on oral and maxillofacial bone regeneration in animal models are summarized in Table 2 [32–36].

**Figure 2:**
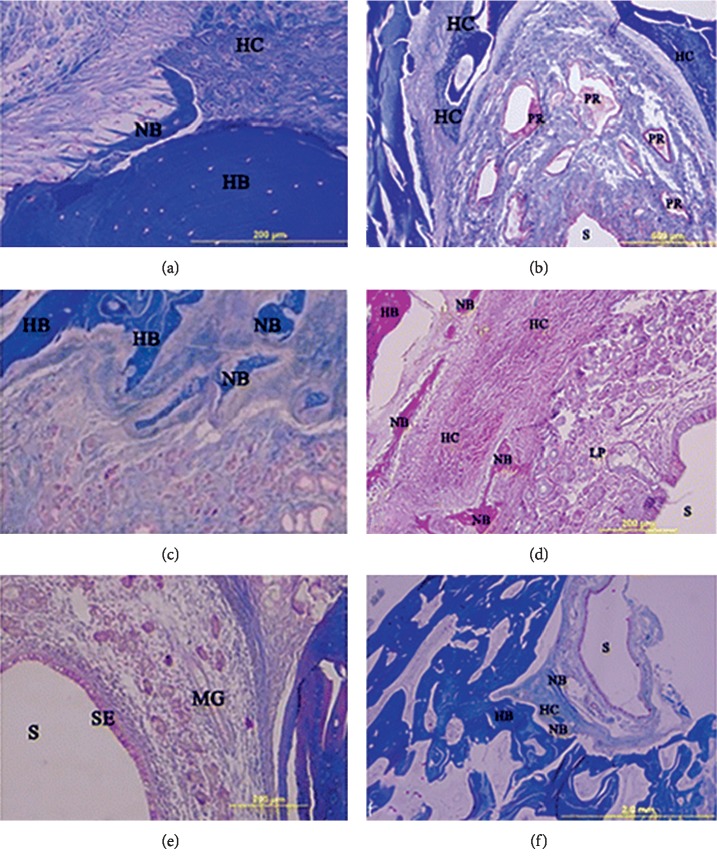
(a) Histological view of newly formed bone at the third month. (b) Cartilage tissue gradually replaced with new bone trabecules in connective tissue at the sixth month. (c) New bone could not be distinguished from the host bone at the ninth month in graft groups. (d) Platelet-rich fibrin (PRF) particles surrounded by compact fibrous capsules at the third month. (e) Newly formed bone was seen between the connective tissue and the host bone at the sixth month. (f) New bone formation is still continuing at the ninth month in PRF groups. S, sinus cavity; SE, sinus epithelium; LP, lamina propria; P, periosteum; PR, PRF remnants; HB, host bone; NB, new bone; MG, mucous glands; ED, edema.

First, PRF alone may enhance bone formation. The first bilateral mandibular molars of eight beagles were extracted, and implants were placed immediately. Then, PRF was placed between socket walls and implants on one side. Six weeks later, the sides with the insertion of PRF showed significantly higher bone area fraction occupancy (BAFO) in histometric results (*p* < 0.05). It indicated that PRF alone promoted bone formation [32]. However, PRF alone has a limited ability for osteogenesis compared with common materials. Twenty-two adult sheep underwent maxillary sinus floor elevation [33]; the filling material used in group I was bovine and autogenous bone mixture, and the filling material used in group II was PRF. New bone formation was seen in group I at the third and sixth months. In group II, new bone formation was observed only at the sixth month. At the ninth month, host bone and new bone could not be distinguished from each other in group I, and bone formation was found to be progressive in group II (Figure 2). Thus, bovine and autogenous bone mixture was better than PRF for maxillary sinus floor elevation, and PRF alone may have a limited effect on osteogenesis.

Second, addition of PRF could improve the osteogenesis ability of other materials. According to a study by Pripatnanont and colleagues [34], addition of PRF to a modified Hyrax device significantly improved the histological and radiological outcomes such as bone volume and bone area in a rabbit model of osteogenic periosteal distraction (OPD) at 4 or 8 weeks (*p* < 0.001). Addition of PRF may result in occupying more space between the original bone surface and the periosteum, thus inducing more neogenesis than the device alone. Mature bone with dense trabecular bone may be related to the growth factors in PRF. However, PRF without a device did not improve the bone quantity than that in the sham group. Therefore, researchers concluded that PRF is just an adjunct therapy for bone regeneration. In another rabbit model of orthodontic relapse [35], addition of advanced PRF (A-PRF) to carbonated hydroxyapatite (CHA) reduced the relapse rate and relapse distance, and this was associated with increased osteoblasts and decreased osteoclasts that were counted histologically.

Third, addition of a drug could improve the osteogenesis ability of PRF, and it is a method to achieve better bone formation by using PRF. A 12-week animal experiment [36] proved that adding aspirin improved the osteogenesis ability of PRF by using a periodontal bone defect model in 15 rats. The result may be related to the structure of PRF/aspirin complex, and SEM showed that PRF had an irregular grid‐like arrangement of loose fibers and pores. In contrast, the PRF/aspirin complex consisted of compact clusters of fibers, and more platelets and leukocytes were observed. Thus, aspirin/salicylic acid could be released from the PRF/aspirin complex in a sustained manner, which could inhibit inflammation and improve the function of mesenchymal cells. On histological evaluation, the proportion of newly formed bone was 38.8 ± 2.6% in the PRF group and 81.1 ± 12.9% in the PRF/aspirin complex group. The volume of newly formed bone was 2.21 ± 0.54 mm^3^ in the PRF group and 4.93 ± 0.88 mm^3^ in the PRF/aspirin complex group, on radiographic examination. It is obvious that new bone in the PRF/aspirin complex group was more than twice of that in the PRF group. The strategies to improve the structure and components of PRF are worthy of further study.

These five animal studies discussed PRF alone, PRF combined with other materials, and PRF/drug mixture. PRF alone may have a limited ability for osteogenesis; therefore, combining PRF with materials or a drug may be a better choice.

## 4. Effect of PRF on Human Oral and Maxillofacial Bone Regeneration

### 4.1. PRF Alone

In almost all published studies (Table 3 [37–55]), opinions about the osteogenic ability of PRF alone have varied. PRF alone was mainly used for the treatment of maxillary sinus augmentation, intrabony defects (IBD), and tooth extraction. Most scientists agreed that PRF alone can improve bone formation, but many scientists suspected this possibility. Besides, PRF was proved to have a limited osteogenic ability compared with common materials.

Research works on maxillary sinus augmentation and IBD treatment revealed good results after PRF application, but lack of control groups was always thought to undermine the conclusions. Two clinical studies, in which PRF was solely used for maxillary sinus augmentation [37, 38], showed that PRF promoted bone gain. One case report described the posttreatment outcomes in a 59-year-old patient in whom the sinus cavity around the implants was full of a dense bone-like tissue, osteocytes were found to be regularly dispersed in the newly formed bone tissue, and osteoblasts were evident on the bone surface [37]. Another prospective study [38] including 27 patients, in whom two types of implants were used, found that residual bone in the sandblasted acid-etched (SA) and hydroxyapatite (HA) groups measured 2.85 mm and 2.68 mm before surgery, but the bone gain was 4.38 mm and 4.00 mm one year later, respectively. Besides, perforation of the sinus membrane may reduce bone formation during sinus elevation [56], but no obvious perforation was seen in this study. PRF was thought to protect the sinus membrane, and thus, PRF alone appears to be suitable for sinus augmentation. In IBD therapy, PRF was found to be beneficial for bone formation. In two clinical studies using PRF in IBD after cystic enucleation, during follow-up on the first, third, and sixth months, all patients showed obvious and gradual radiographic osseous regeneration. Radiographically, complete bone regeneration was seen in all patients within six postoperative months [39, 40]. Three case reports including 18 patients used PRF alone to treat IBD caused by a primary periodontal lesion [41], periradicular lesions of endodontic origin [42], and endoperio lesion in an immature right mandibular first premolar [43], and they showed successful complete bone fill, faster than routine treatment. Nagaveni et al. [43] even underlined that bone fill was similar to adjacent normal teeth on the radiograph.

There are more disputes regarding whether PRF alone could promote bone formation in control trials. In controlled trials of tooth extraction, a greater bone density was achieved in the PRF group compared with the blank control group [44–46] or the PRP group [47]. Singh and his colleagues reported on 20 patients who were treated with PRF in one socket and no PRF in another socket; at the 12th week, all patients showed trabecular bone formation and higher gray level value at the PRF site (146.9) than the non-PRF site (123) [44]. Another report confirmed the effectiveness of PRF by histological analysis of 28 patients who were treated with or without PRF (*n* = 14 each). New bone formation was abundant in the PRF group [45]. A total of 30 patients received PRF in one extraction, and the other region was without PRF, and the bone density value evaluated by CBCT at 24 h and 3 months in the socket regions in the PRF group was 319.79 and 564.76, respectively, while in the blank control group, the corresponding value was 194.82 and 295.87, respectively (*p* < 0.05) [46]. PRF is similar to PRP, but it is convenient and cheaper than PRP; therefore, it makes sense to compare PRF and PRP in regeneration. In a report of 20 patients who received PRF in the right side and PRP in the left side, the mean values of bone density were higher in the PRF groups. The *p* value was 0.000 on digitalized OPG images [47]. However, other three tooth extraction trials showed no significant difference in bone formation between PRF and blank control groups [48, 49] or between PRF and PRP on radiographic evaluation [50]. In controlled trials of inflammation-induced bone defect, two randomized controlled trials (RCTs) [51, 52] proved that PRF enhanced bone fill in IBD compared with open flap debridement (OFD) only. In a study, 13 patients with 26 IBD sites were divided into the PRF or OFD alone groups; the percentage of bone fill in PRF sites was 45.18% ± 7.57%, while the percentage of bone fill in OFD sites was 21.6% ± 9.3% (*p* = 0.001) on radiographic evidence at 12 months [51]. In another study, 17 patients with 54 IBD sites were divided into the PRF or OFD alone group, and PRF (46.14% ± 11.39%) caused a greater percentage change in IBD depth than at the OFD sites (15.76% ± 18.77%) at 9 months (*p* < 0.001) [52] radiologically. However, a controlled trial [53] revealed that PRF did not contribute to bone regeneration radiographically.

When compared with common materials, PRF may have no obvious advantage. Two RCTs revealed that HA [54] and autogenous bone grafting (ABG) [55] have greater osteogenic ability than PRF. Hence, PRF alone has an unstable effect on osteogenesis among experiments; PRF may enhance osteogenesis, but it also has disadvantages during bone regeneration such as lack of rigidity and faster degradation [57]. Thus, it is necessary to develop improved approaches for better application. Herein, we will review the common strategies including PRF combined with materials, PRF combined with drugs, and a new-type PRF here.

### 4.2. PRF Combined with Bone Graft Materials

In the case reports shown in Table 4 [58–65], 28 patients were treated by PRF combined with other bone graft materials. All patients showed bone regeneration on histomorphometric or imaging examination. The disease types included periapical inflammatory lesion with bony defect [58, 59], IBD [60, 61] maxillary sinus augmentation [62–64], and extraction of the molar teeth [65]. Moreover, Pichotano et al. [63] filled the right sinus with PRF, deproteinized bovine bone mineral (DBBM), and CM, but they filled the left side with DBBM and CM in a patient for maxillary sinus augmentation, and they found higher proportion of bone formation when using PRF on histomorphometric analysis (2,118,102 mm^3^ and 975,535 mm^3^, respectively). However, a small sample size and lack of a control group undermined the conclusion of these studies. Also, they could not clearly explain the osteogenesis effect of PRF or bone graft materials.

Some controlled trials [66–74] showed better osteogenesis effect when using PRF combined with bone graft materials compared with PRF or materials alone, as shown in Table 4. In most experiments, materials were added to PRF [66–69], and they aimed at promoting effective space maintenance and osteoconductive effect or providing cells and factors. PRF can also improve the properties of graft materials [70–74] by providing cytokines, platelets, leukocytes, and circulating stem cells. The results of a study [73] suggested that PRF can act as a delivery system for graft particles in maxillary sinus floor augmentation. The time required for new bone formation is closely linked to the graft volume. Fibrin helps to prevent dispersion of the Bio-Oss® particles; as a result, less sinus graft material is needed to obtain sufficient vertical height of the material for placement of implants.

However, another five controlled trials [75–79] showed that PRF may not enhance bone formation when combined with other materials. Sezgin et al. [75] thought that the use of ABBM might have masked the positive effects of PRF. Turkal et al. [76] thought that EMD or PRF is not physically rigid, and therefore, these materials are not able to provide effective space maintenance. This finding may explain why PRF did not cause additional bone gain with EMD.

Finally, based on the above studies, we summarize that the materials enhanced the osteogenesis ability of PRF, thus emphasizing the benefit exerted by these materials. Additionally, we also summarize some improved protocols for better application of these materials.

#### 4.2.1. Synthetic Materials

HA constitutes 60%–70% of bone [80]. Similarity of HA to bone makes it superior over other calcium phosphates [81], and HA is biocompatible, osteoconductive, and bioactive [82]. In two case reports [58, 59], a periapical inflammatory lesion with bony defect was filled with a combination of PRF and HA bone graft crystals, and the authors found that HA was replaced by new bone radiographically. HA can also be fabricated into porous scaffolds, which are conducive to cell attachment, migration, and differentiation [83, 84]. An RCT [66] proved that the addition of a porous HA graft to PRF enhanced the percentage of bone fill.

It has been reported that ionic products released by bioactive glasses (BG) can stimulate bone formation [85]. Besides, an HA-like surface layer will form when BG is in biological fluids, which enhances the binding force to bone [86]. It was proved that adding BG enhanced the degradability and bioactivity for bone bonding of HA [87]. Based on these results, HABG and PRF composites were used for IBD, and complete healing of the defect was seen radiographically in a case report [61].

#### 4.2.2. Natural Materials

Common bovine‐derived xenografts, such as DBBM, bovine porous bone mineral (DPBM), and demineralized bone matrix (DBM), are widely used in oral and maxillofacial bone regeneration. The drawbacks of obtaining autografts can be avoided, and long-term results similar to those of autografts have been obtained by applying bovine‐derived xenografts [88]. Moreover, PRF is inadequate for space maintenance during bone regeneration; therefore, adding mineralized and rigid materials can enhance the osteoconductive and space-maintaining effect of PRF [67, 68]. In five case reports, DBBM and PRF composites were used for IBD [60], maxillary sinus augmentation [62–64], and tooth implantation [65], and new bone formation was noted in all cases. A clinical controlled study on treatment of IBD proved that adding DPBM improved the osteogenesis ability of PRF [67], and defect fill was 4.06 ± 0.87 mm at the buccal site and 3.94 ± 0.73 mm at the lingual site in the PRF-BPBM group, while defect fill was 2.21 ± 0.68 mm at the buccal and 2.06 ± 0.64 mm at the lingual site in the PRF group. Besides, DBM has BMP, which is released during the demineralization process, and some of the BMPs [89] could stimulate the process of stem cell differentiation. Practically, using PRF combined with DBM indeed filled the IBD more effectively than PRF alone [68], and linear bone growth (LBG) and percentage of bone fill (%BF) were higher in the PRF/IBD complex group (*p* < 0.05).

The amnion membrane was harvested from the sac that encloses the embryo. It is elastic and thin. The amnion membrane consists of pluripotent stem cells and all types of growth factors [90], such as EGF, NGF, VEGF, and TGF-*β*1, which could explain how it enhances the osteogenesis ability of PRF in the treatment of grade II furcation defects [69]. Use of the combination of PRF/amnion membrane caused more bone formation at 6 months of growth. The mean difference in percentage change in radiographic linear bone growth was 15.08 ± 6.41 (*t* = 2.349 and *p*=0.026), while the mean difference in volumetric bone gain at 6 months was 1.75 ± 0.57 (*p*=0.005).

### 4.3. PRF Combined with a Drug

RCTs [91–94] (Table 5) have revealed that adding drugs promoted the osteogenesis effect of PRF. Drugs used in these trials were alendronate (ALN) [91, 92], rosuvastatin (RSV) [93], and atorvastatin (ATV) [94].

Two RCTs [91, 92] showed that when treating furcation defects, PRF + ALN enhanced bone formation than PRF alone (*p* < 0.05). ALN enhanced bone formation by itself. First, it has high binding affinity to HA crystals, and it prevents their dissolution [95]. Moreover, it acts as an inhibitor of osteoclastic bone resorption [96].

An RCT [93] conducted by Pradeep et al. found that combining RSV, PRF, and HA exerts synergistic effects, amplifying their role in the treatment of furcation defects, thus achieving a greater amount of bone fill when RSV was added to a mixture of PRF and HA. In another RCT [94] conducted by Martande et al., 1.2% ATV was added to PRF, and ATV augmented the regenerative potential of PRF alone in periodontal IBDs. These two phenomena may be related to a mechanism that caused statin‐induced osteoblast differentiation by boosting BMP‐2 gene expression and secretion [97].

### 4.4. A New-Type PRF

To improve the components and structure of PRF or to overcome some problems and limitations during application, a new-type PRF was produced (Table 6 [98–102]). We present the applications of A-PRF, I-PRF, and T-PRF here.

#### 4.4.1. Using a Different Centrifugal Force

The components and structure of PRF could be modified by using a different centrifugal force [103]. By lowering the centrifugal force, advanced PRF (A-PRF) and injectable PRF (I-PRF) can be produced. A-PRF has more neutrophils, which can stimulate monocytes to differentiate into macrophages and release more growth factors to promote bone regeneration [104–106]. I-PRF has lower consistency than PRF, and it mainly improves the difficulty in combination with bone biomaterials [107]. In two case reports, A-PRF and I-PRF were combined with individualized 3D planned titanium mesh [98] and Bio-Oss, respectively [99]. They showed significant bone regeneration. Lorenz et al. [98] thought that their method reduced the surgery time, postoperative pain, and healing time compared with an autograft, and it achieved the goal of three-dimensional bone regeneration. In the report by Lei et al. [99], it was found that a 3D scaffold could accurately fill the bone defect and maintain a stable repair space, and I-PRF could accelerate the solidification of A-PRF and shorten the molding time of A-PRF. Moreover, A-PRF could enhance the binding force between Bio-Oss and improve the plasticity of materials, and I-PRF could further consolidate this binding force during regeneration.

#### 4.4.2. Using Titanium Tube during Centrifugation

To avoid the health hazard caused by silica particles in glass tubes during centrifugation of blood, Tunalı et al. produced titanium-prepared PRF (T-PRF) by using a titanium tube instead of a glass tube. T-PRF has a tighter and thicker fibrin structure and a longer release time of growth factors than PRF, which may be more conducive to tissue regeneration [108, 109].

The use of T-PRF alone in 39 patients undergoing maxillary sinus elevation operations achieved successful clinical and histomorphometric results. Bone formation in the T-PRF group was accelerated to 4 months compared to that with allografts, according to the histological results [101]. Another research [102] proved that T-PRF has the same bone regeneration effect as PRF in IBD, which may also suggest that the use of a titanium tube does not alter the effect of PRF.

## 5. Shortcomings in the Application of PRF

Failure of bone regeneration after using PRF may be related to some avoidable issues; therefore, it is necessary to discuss these issues.

### 5.1. Preparation Process

The preparation process of PRF is controversial. First, the use of different experimental animals may lead to different experimental results or even failure. For example, stable fibrin polymers cannot be collected from rabbits, except for blood collection from the heart. Therefore, some scholars have suggested to avoid rabbits and give priority to large animals such as beagles for blood collection [110]. Moreover, when simulating human anatomical, physiological, and biomechanical environments, large animals are better than rodents [111].

Second, there are problems during centrifugation. Silica particles in glass tubes may remain suspended in PRF causing health risks [112]; therefore, some authors suggested the use of titanium tubes [109]. Many researchers inaccurately reported the relative centrifugal force (RCF) values, thus leading to different and confusing products. As a result, some authors suggested the use of necessary parameters during centrifugation in future studies [113].

Third, in clinical practice, the characteristics of PRF may be affected by the patient's age, systemic diseases (such as thrombocytopenia, hemorrhagic disease, and diabetes), nutrition status, environmental or ethnic differences, autoimmunity, and genetic susceptibility [114]. For example, Yajamanya et al. found that the fibrous protein in PRF changed with age: the density decreased and it became loose, and the number of platelets and white blood cells also decreased [115]. The optimal ratio of cytokines for bone regeneration is also controversial. In a study, growth factors (IGF-1, PDGF, TGF-*β*, and FGF) in PRF may reduce ALP synthesis through an antagonistic action, thus reducing bone mineralization [48]. Ohshima et al. found that TGF-*β* and VEGF are involved not only in tissue regeneration but also in tissue degradation [105, 116, 117]. Thus, how to adjust the ratio of cytokines remains to be studied.

### 5.2. Experimental Design

There are also some shortcomings in the experimental design of bone tissue regeneration by PRF. The limitations of clinical research include small number of samples, failure of long-term observation, and lack of histological assessment. Different studies used different experimental groups and control groups, and experimental evaluation methods were also different, which also led to different conclusions. Besides, more RCTs are needed to study various other factors during PRF application, such as type and amount of grafting materials [37]. Therefore, in the future study, the preparation process of PRF and the experimental design for the regeneration of oral and maxillofacial bone tissue with PRF need to be further improved.

## 6. Conclusion

Research findings indicate that PRF as a bone graft material is a promising treatment option for oral and maxillofacial bone regeneration. PRF has been proved to improve proliferation, differentiation, migration, and mineralization of cells during bone formation, and the effects vary by the cell type. However, PRF alone has an unstable effect on osteogenesis. In this paper, we have discussed the improved approaches, including PRF combined with materials, PRF combined with drugs, and a new-type PRF, used in many preclinical and clinical studies related to oral and maxillofacial bone regeneration. Finally, we have also discussed some shortcomings in PRF application, and we hope that future studies will optimize the preparation process and the experimental design of PRF, thus leading to better bone quality.

## Figures and Tables

**Table 1:** Summary of effects of platelet-rich fibrin on stem cells.

Cell type	Intervention	Outcome	Reference
Rat GSPCs	PRF	Increased CBF-*α*1 expression	[27]

Rat PDLSCs	Cell culture: PRF; surgical procedure: implanted PRF membrane and rat PDLSCs	Increased cell proliferation and enhanced ALP activity and OC, RUNX2, and BSP mRNA and protein levels; promoted expressions of COL1A, Opn, and RUNX2 with enhanced new alveolar and mandibular bone	[28]

Human osteoblasts	PRF	Enhanced lactate dehydrogenase test values, cell growth, proliferation, and alkaline phosphatase activity	[23]

Human periodontal progenitors: PDL, DF, AB	PRF with 10% FBS	Increased cell proliferation, migration, alkaline phosphatase, alizarin red staining, and expression of RUNX2, but reduced expression of MGP	[29]

Human PDLSCs	PRF	Induced cell proliferation but decreased ALP activity and gene expressions of BSP and OCN	[30]

DF: dental follicle progenitors; FBS: fetal bovine serum.

**Table 2:** Summary of animal studies on platelet-rich fibrin in oral and maxillofacial bone regeneration.

Animal model	Intervention	Outcome	Reference
Implantation after tooth extraction in dogs	Implants with or without PRF	The presence of PRF resulted in higher BAFO histologically	[32]

Maxillary sinus floor elevation	Bovine and autogenous bone mixture or PRF	The bovine and autogenous bone group yielded better histological results than the PRF group	[33]

OPD in rabbits	Device + PRF, device, PRF, and sham	The device + PRF group presented the highest percentages of bone volume and bone area histologically and radiologically	[34]

Orthodontic relapse in rabbits	Control group, CHA, and CHA-A-PRF	Relapse rate and relapse distance were lower (*p* < 0.05) in CHA-A-PRF. The number of osteoblasts was higher and that of osteoclasts was lower in CHA-A-PRF histologically	[35]

Periodontal bone defect in rats	No treatment, PRF, or PRF/aspirin complex.	New bone in the PRF/aspirin complex group was more than twice of that in the PRF group histologically	[36]

**Table 3:** Summary of clinical effects of platelet-rich fibrin alone on oral and maxillofacial bone regeneration.

Patient number (age/range)	Disease type	Intervention	Follow-up	Outcome	Reference
1 (59 years)	Atrophy of maxillary posterior edentulous areas	PRF was filled after maxillary sinus floor elevation	6 m	Bone formation was seen radiologically and histologically	[37]
27 (29−74 years)	Atrophy of maxillary posterior edentulous areas	PRF was filled after maxillary sinus floor elevation	12 m	Bone gains were 4.38 mm and 4 mm in the SA and HA groups radiologically	[38]
10 (23−45 years)	IBD	PRF	6 m	Complete bone formation was seen radiologically	[39]
20 (20−55 years)	IBD	PRF	6 m	Complete bone formation was seen radiologically	[40]
2 (24 years and 32 years)	IBD	PRF	9 m	Considerable bone fill was seen radiologically	[41]
15 (20−50 years)	IBD	PRF	6 m	Complete bone fill was seen radiologically	[42]
1 (12 years)	IBD	PRF	6 m	Complete bone fill was seen radiologically	[43]
20 (18−50 years)	Extraction of teeth (40 sites)	PRF	3 m	Increased bone density radiologically	[44]
28 (20−40 years)	Extraction of teeth	PRF	3 m	Enhanced bone gain histologically	[45]
30 (20−50 years)	Extraction of teeth (60 sites)	PRF	30 m	Enhanced bone density histologically	[46]
20 (18−28 years)	Extraction of teeth (40 sites)	PRF or PRP	4 m	Increased bone density radiologically	[47]
20 (19−34 years)	Extraction of teeth (40 sites)	PRF	3 m	No significant difference in bone density	[48]
34 (18−40 years)	Extraction of teeth (68 sites)	PRF	6 m	No significant difference in bone quantity	[49]
30 (18−30 years)	Extraction of teeth	PRF or PRP	6 m	No significant difference in bone density	[50]
13 (35−55 years)	IBD (26 sites)	PRF	12 m	Increased bone fill percentage radiologically	[51]
17 (20−30 years)	IBD (54 sites)	PRF	9 m	Increased IBD depth change radiologically	[52]
15 (28−44 years)	Horizontal bony defects (45 sites)	PRF	9 m	No significant difference in RCH radiologically	[53]
40 (17−36 years)	Extraction teeth	PRP or PRF or HA	6 m	Lesser bone density values were seen in PRP, PRF, and control site at 1, 2, and 6 months than at the HA site radiologically	[54]
20 (30−55 years)	IBD	PRF or ABG	9 m	ABG showed greater RBF as compared with PRF	[55]

RCH: relative bone crest height; RBF: radiographic bone fill.

**Table 4:** Summary of clinical effects of platelet-rich fibrin combined with materials in oral and maxillofacial bone regeneration.

Patient number (age/range)	Disease type	Intervention	Follow-up	Outcome	Reference
1 (45 years)	Periapical bony defect	PRF and HA	24 m	New bone replaced HA almost completely radiographically	[58]
3-case report (19−24 years)	Periapical bony defect	PRF and HA	12 m	New bone replaced HA radiographically	[59]
1 (35 years)	IBD	PRF and Bio-Oss	1 8 m	Increased radiographic bone fill	[60]
1 (25 years)	IBD	PRF and HABG	12 m	Complete healing of the defect radiographically	[61]
4-case report (43−59 years)	Atrophy of maxillary posterior edentulous areas	PRF and DBBM were filled after maxillary sinus augmentation	7 m or 10 m	Mean percentage of new bone was 34.5% ± 5.7% histomorphometrically	[62]
1 (59 years)	Atrophy of maxillary posterior edentulous areas	PRF and DBBM were filled after maxillary sinus augmentation	8 m	More newly formed bone than by using DBBM alone histomorphometrically	[63]
14-case report (—)	Atrophy of maxillary posterior edentulous areas (30 sites)	PRF and Bio-Oss were filled after maxillary sinus augmentation	6 m	Mean vertical bone height gain was 10.12 mm radiographically	[64]
1 (38 years)	Extraction of teeth	PRF and Bio-Oss	6 m	New bone regeneration around the neck of the implant radiographically	[65]
57 (mean age: 39.7 years)	IBD (90 sites)	Group I : PRF + OFD; group II : PRF + HA + OFD; group III : OFD	9 m	Percentage of mean bone fill radiographically in group I was 56.46% ± 9.26%, in group II was 63.39% ± 16.52%, and in group III was 15.96% ± 13.91%	[66]
17 (mean age: 44 ± 9 years)	IBD (34 sites)	PRF or PRF-BPBM combination	6 m	Defect fill was greater in the PRF-BPBM group radiographically	[67]
36 (30−50 years)	IBD	Group I : PRF + DBM; group II : PRF; group III : OFD	9 m	Significant improvement in LBG and %BF was found in group I radiographically (*p* < 0.05)	[68]
15 (mean age: 36.1 years)	Grade II furcation defects (30 sites)	Group I : PRF and amnion membrane; group II : PRF	6 m	More volumetric bone gain and radiographic linear bone growth was seen in group I	[69]
10 (20−50 years)	IBD (20 sites)	PRF and bioactive glass putty (test group) or bioactive glass putty alone (control group)	9 m	The radiographic bone fill from baseline at the control site was 5.70 ± 1.64 and that at the test site was 7.10 ± 1.37 (*p* < 0.05)	[70]
16 (25−65 years)	Class II furcation defects (20 sites)	PRF and BCCG (test sites) or BCCG alone (control sites)	6 m	More percentage defect fill was seen in the test group (*p* < 0.05). Increase in radiographic bone density at the furcation defect in the test group (*p*=0.036)	[71]
20 (27−45 years)	IBD (40 sites)	Group I : BG + PRF; group II : BG alone	6 m	More defect depth reduction was seen in group I (*p* < 0.05) radiographically	[72]
6 dogs (adult)	Atrophy of maxillary posterior edentulous areas (12 sites)	Group I : PRF and Bio-Oss; group II : Tisseel and Bio-Oss was filled after maxillary sinus augmentation	6 m	The mean new bone formation rate was 41.8 ± 5.9% in group I, and in group II, it was 31.3 ± 6.4% (*p* < 0.05) radiographically	[73]
12 (43−63 years)	Atrophy of maxillary posterior edentulous areas (38 sites)	DBBM + L‐PRF (test) or DBBM alone (control) was filled after maxillary sinus augmentation	4 m (test), 8 m (control)	Newly formed bone in the test group was 44.58% ± 13.9% and that in the control group was 30.02% ± 8.42%; *p*=0.0087 on histological evaluation	[74]
15 (38−61 years)	IBD (30 sites)	ABBM (control group) or ABBM-PRF combination (test group)	6 m	Defect fill was not statistically different radiographically	[75]
28 (age ≥18)	IBD (56 sites)	IBDs were randomly treated either with EMD or with EMD + PRF	6 m	Defect fill was not statistically different radiographically	[76]
10 (—)	IBD (20 sites)	Group I : DFDBA; group II: mixture of PRF with DFDBA	6 m	Mean defect fill and mean defect resolution were not statistically different radiographically	[77]
13 (35−65 years)	Atrophy of maxillary posterior edentulous areas (26 sites)	DBBM and PRF mixture (test) or DBBM (control) was filled after maxillary sinus augmentation	6 m	Newly formed bone was similar (*p* > 0.05)	[78]
22 (6−28 years)	Alveolar cleft (13 unilateral and 9 bilateral)	Group A: autogenous bone grafts; group B: autogenous bone grafts with PRF	6 m	Percentages of newly formed bone were similar (*p* > 0.05)	[79]

**Table 5:** Summary of clinical effects of platelet-rich fibrin combined with drugs in oral and maxillofacial bone regeneration.

Patient number (age/range)	Disease type	Intervention	Follow-up	Outcome	Reference
72 (30−35 years)	Furcation defects	PRF (group II) or PRF + 1% ALN (group III)	9 m	PRF + 1% ALN showed a greater percentage of radiographic defect fill (56.01% ± 2.64%) compared with the PRF group (49.43% ± 3.70%) (*p* < 0.001)	[91]

20 (38−56 years)	Furcation defects (40 sites)	PRF group or PRF+1% ALN group	6 m	More mean reduction in radiographic bone defect volume for PRF + ALN (11.98 ± 4.13 mm^3^) than the PRF group (8.65 ± 3.84 mm^3^) (*p* < 0.05)	[92]

105 (25−55 years)	Furcation defects	Placebo gel (group I), PRF + HA (group II), or 1.2 mg RSV gel + PRF + HA (group III)	9 m	A greater percentage of radiographic mean bone fill was found in group II (54.69% ± 1.93%) compared with group III (61.94% ± 3.54%) and group I (10.09% ± 4.28%) (*p* < 0.05)	[93]

96 (30−50 years)	IBD	PRF or PRF + 1.2% ATV	9 m	PRF + ATV caused a greater percentage radiographic defect depth reduction compared with PRF alone (*p* < 0.05)	[94]

**Table 6:** Summary of clinical effects of modified platelet-rich fibrin in combination in oral and maxillofacial bone regeneration.

Patient number (age/range)	Disease type	Intervention	Follow-up	Outcome	Reference
1 (61 years)	Bony defect within the mandible	Solid A-PRF and liquid I-PRF together with an individualized 3D planned titanium mesh	8 m	New bone originated from the residual bone on histological analysis, while oral function complete rehabilitation and restoration	[98]

1 (36 years)	IBD	A-PRF and I-PRF, which were mixed with Bio-Oss and packed onto the 3D replica	15 m	Significant radiographic 3D alveolar bone fill	[99]

18 (42−69 years)	Atrophy of maxillary posterior edentulous areas	T-PRF or allografts	6 m	Bone formation after 6 months of allografts was achieved in the T-PRF group at only 4 months radiologically and histologically	[101]

38 (20−55 years)	IBD (90 sites)	PRF or T-PRF	9 m	No statistically significant difference in defect depth reduction	[102]
